# Factors associated with the adoption of extreme weight control behavior by non-obese adolescents: a secondary analysis

**DOI:** 10.1186/s12887-023-04299-1

**Published:** 2023-09-14

**Authors:** Eunha Jeong

**Affiliations:** Pungnap Middle School, Seoul, Republic of Korea

**Keywords:** Weight perception, Thinness, Adolescent, Weight control behaviors, Weight loss, Risk factors, Non-obese, Health education

## Abstract

**Background:**

Misperceptions about obesity is common among adolescents. Adolescents who overestimate their body size tend to indulge in extreme weight control behaviors. However, little is known about the factors involved in the adoption of extreme weight control behavior (EWCB) by non-obese adolescents who are mistaken for being overweight. This study identified factors associated with unhealthy behaviors among normal/underweight high school students who overestimate their body image and attempt to lose weight.

**Design:**

A secondary analysis of nationally representative data from the Korea Youth Risk Behavior Survey focused on adolescents who attended vocational and academically oriented high schools.

**Methods:**

The analysis included data from 4,286 non-obese respondents (15–18 years) who overestimated their body weight. Of them, 2,887 were girls (66.5%), while 1,399 were boys (33.5%). Multiple logistic regression was used to investigate risk factors for EWCB by sex. A statistical analysis reflecting strata, clusters, and weights of the complex sampling design was adopted.

**Results:**

Of the respondents, 674 (23.3%) girls and 162 (11.5%) boys reported EWCB. For both sexes, vocational high school attendance and depression were significantly influenced by EWCB. EWCB was linked to perceived stress in girls and living in a big city in boys.

**Conclusions:**

The findings suggest the importance of providing quality health education, including that for non-obese adolescents, in school obesity prevention programs along with the expansion of tailored intervention programs based on sex, following a consideration of the characteristics of high schools as well as individuals.

## Background

Misperceptions of being overweight are common among adolescents. A national survey conducted in Canada reported that 15.8% of normal-weight female adolescents and 6.0% of normal-weight male adolescents perceived themselves as overweight [1]. These rates are even higher in South Korea, where one study found the respective percentages to be 33.7% and 20.9% [2]. Such high numbers are concerning, as adolescents who overestimate their body size are prone to extreme weight control behaviors (EWCB) [3], which thus highlights the need for evidence-based educational interventions and other relevant measures aimed at the promotion of healthy behaviors in this group.

EWCB includes induced vomiting and drug use, as well as fasting and using a food substitute for weight loss [4–6]. The adoption of EWCBs, such as fasting, post-meal vomiting, and drug use, results in negative physical and mental health outcomes [7] and increases the risk of eating disorders [8]. A longitudinal study demonstrated that the attempt of dieting or EWCB in late adolescence can be followed into adulthood [9].

While the overestimation of one’s weight is strongly related to EWCB [3, 10], not all individuals with this misperception take unhealthy actions to lose weight [11]. EWCBs such as fasting, ingesting laxatives, and consuming “diet” foods are associated with various negative outcomes, especially for adolescents [9]. Particularly, underweight adolescents or those with normal weight who engage in extreme weight-loss behaviors are at risk of undernutrition, which can hinder their growth and maturation [12]. In this context, body image distortion appears to be a major factor [13]. However, there is still a general lack of evidence on other issues that may influence extreme weight-loss behaviors in adolescents with overweight misperceptions.

Previous studies have primarily focused on the identification of weight-related factors such as actual BMI and subjective body type recognition as the primary related factors for EWCB [5, 10, 14, 15]. Alternatively, adolescents were considered as a single group to reveal the related factors of EWCB. EWCB-related socio-demographic factors such as sex, age, school sex-composition, school type, geographic area, perceived economic status, and/or parents’ final educational background [4, 5, 7, 15–17], and psychological factors such as self-rated health, perceived stress, and/or experience of depression [5–7, 14, 15, 17] were identified in domestic and overseas studies.

However, in the case of adolescents in Korea, the purpose of weight control is not to promote health, but appearance [18]. Adolescents who perceive themselves as overweight attempt weight control or adopt EWCBs even though weight control is unnecessary [19], which reveals the seriousness of this problem.

Therefore, the purpose of this study is to identify factors related to the EWCBs of adolescents who perceive themselves as overweight despite being underweight or of normal weight by using relevant data from the nationally representative Korea Youth Risk Behavior Survey (KYRBS). The primary research question was “what factors affect EWCB among normal/underweight adolescents who overestimate their body image?”.

## Methods

This study conducted a secondary data analysis from the 15^th^ wave of the KYRBS, as collected in January 2019 by the Korea Centers for Disease Control and Prevention (KCDC). These data were publicly released in January 2020. The KYRBS is an annually implemented, self-reported, cross-sectional, and representative online survey of Korean adolescents. The first wave was conducted in 2005. As arranged by the KCDC, the survey questionnaire consists of 105 items across 15 domains of health behavior (e.g., obesity, weight control, and mental health) [19]. Previous estimates have demonstrated that the KYRBS questionnaire is of good validity [20]. To minimize the sampling error, the sample was extracted via three steps (population stratification, sample allocation, and sampling). Specifically, the survey targeted 60,100 anonymous middle and high school students (grades seven through 12), and thereby obtained 57,303 responses (95.3% participation rate) [19].

**Fig. 1:**
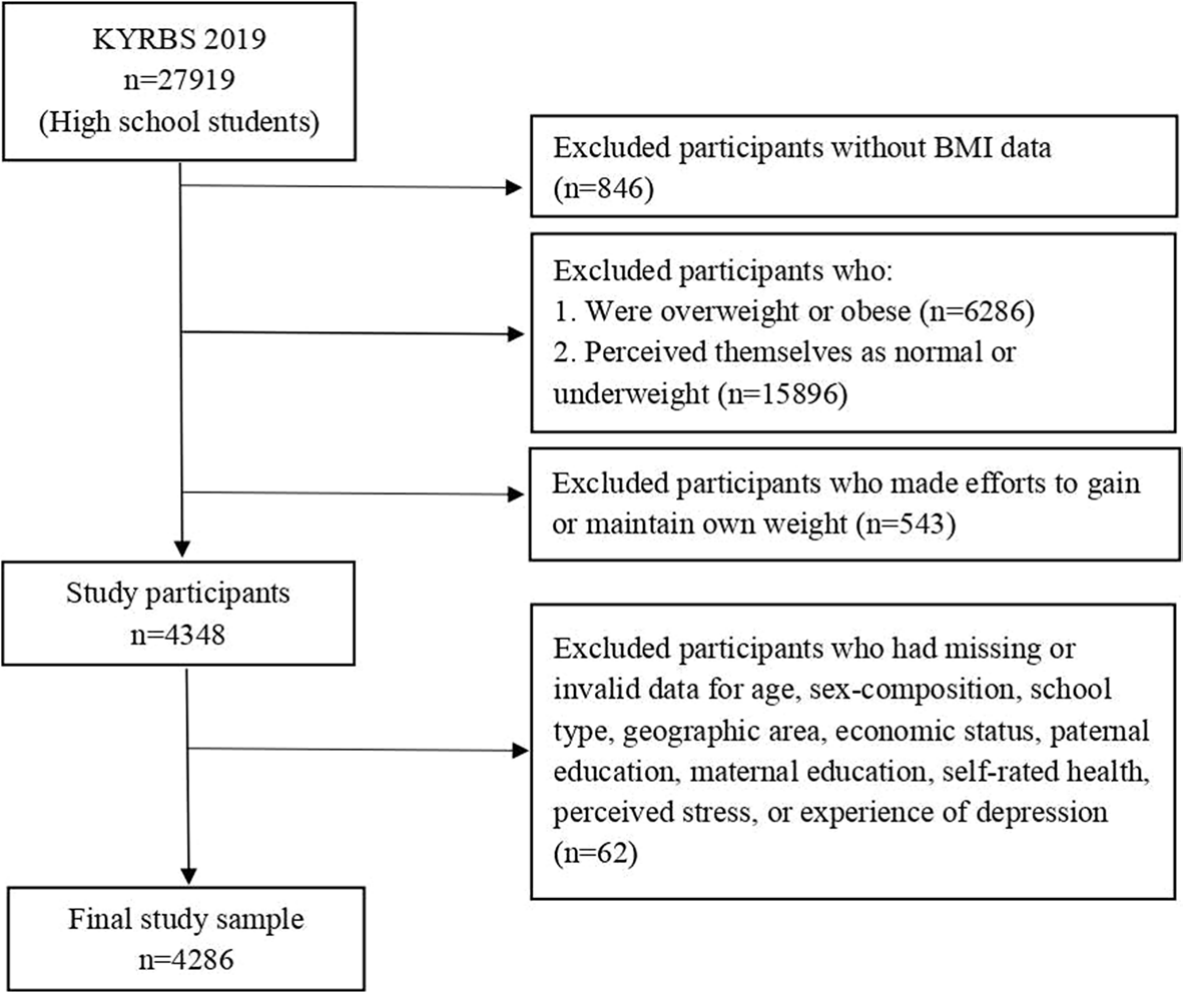
Flowchart illustrating the sample selection process

As mentioned, this study analyzed data from high school students (*n* = 27,919) surveyed during the 15^th^ KYRBS. Of these respondents, 4,286 were underweight or of normal weight but perceived themselves as overweight or obese. Figure 1 illustrates a flowchart of the sample selection process.

The outcome variable EWCB was determined based on “yes” responses to engagement in any of the following actions over the previous 30 days for the purpose of weight control: (a) “fasting (skipping meals for ≥ 24 h),” (b) “using laxatives or diuretics,” (c) “self-induced vomiting,” (d) “taking diet pills/oriental medicine,” (e) “eating only one type of food, such as grapes, eggs, or milk” (known as the one-food diet), and (f) “eating food substitutes (e.g., powder or other special drinks)” [4, 5, 19]. A composite measure of EWCB was created as a binary variable, which indicated whether participants had engaged in at least one of the behaviors over the past month [4, 21].

On the basis of previous studies, it can be discerned that EWCB may be influenced by a variety of sociodemographic factors (e.g., sex, age, school sex-composition, school type, geographic area, perceived economic status, and/or parents’ final educational background) and psychological characteristics (self-rated health, perceived stress, and/or experience of depression) [4–7, 14–17]. In this study, sex (i.e., boy, girl) was used as the primary independent variable, and age, which ranged from 15 to 18 years, was used as a continuous variable. Under demographic information, sex composition (i.e., single-sex, co-ed), school type (i.e., general high school, vocational high school), geographic area (i.e., big city, mid-size/small city, rural area), perceived economic status (i.e., high, middle [mid-high/middle/mid-low], low), and parents’ educational background (i.e., middle school graduate or less, high school graduate, college graduate or higher, and unknown) were considered.

Self-rated health was assessed based on answers to the question, “How would you rate your health?” Responses were categorized into three groups, including “good” (very healthy, healthy), “fair” (fair), and “bad” (very unhealthy, unhealthy). Perceived stress was evaluated based on answers to the question, “How much stress do you usually feel?” Responses were divided into two groups, including “high” (very much, much) and “low” (a little, seldom, never). Finally, experiences of depression were evaluated based on answers to the question, “Have you experienced sadness or despair strong enough to interrupt daily life for more than 2 weeks within the last 12 months?” Here, responses were provided by the indication of “yes” or “no.”

All statistical analyses were conducted using SAS version 9.4 (SAS Institute Inc., Cary, NC, USA), and a *p*-value < 0.05 was considered statistically significant.

Complex sampling methods were employed during the creation of the nationally representative database. Therefore, this study conducted all the statistical analyses in consideration of strata, clusters, and weights of the complex sampling design and validly treated the missing data [19].

Descriptive statistics were used to describe the basic characteristics of the study participants by sex, and mean, standard error (SE) or numbers, and percentages were reported for each variable. Chi-square tests for categorical variables and independent t-tests for continuous variables were used to compare the characteristics of sex-based subjects with and without EWCB. A multiple logistic regression was conducted to explore the associated factors for EWCB within the survey subpopulation. Moreover, the analysis was disaggregated by sex to identify differences in any associations therein. The results are discussed below as odds ratios (ORs) and 95% confidence intervals (95% CIs).

## Results

Table 1 lists the sociodemographic and psychological characteristics of the study sample. Of the 4,286 analyzed respondents, 2,887 were girls (66.5%), while 1,399 were boys (33.5%). As for sex differences, more girls reported high perceived stress (36.9%) and the experience of depression (24.8%) than boys (12.7%; 9.1%).

**Table 1:** Sociodemographic and psychological characteristics of the study participants by sex (*N* = 4286)

Variables	Categories	Total	Girls (*n* = 2887)	Boys (*n* = 1399)
		M ± SE or n (%)	M ± SE or n (%)	M ± SE or n (%)
Age (years)		16.52 ± 0.02	16.50 ± 0.02	16.57 ± 0.03
Sex- composition	Single-sex	1821 (43.6)	1275 (30.4)	546 (13.2)
	Co-ed	2465 (56.4)	1612 (36.1)	853 (20.3)
School type	General high	3580 (84.9)	2436 (57.2)	1144 (27.7)
	Vocational high	706 (15.1)	451 (9.3)	255 (5.8)
Geographic area	Big city	2181 (49.8)	1490 (33.8)	691 (16.0)
	Mid-size/Small city	1844 (45.8)	1220 (29.9)	624 (15.9)
	Rural area	261 (4.4)	177 (2.8)	84 (1.6)
Economic status	High	1364 (32.5)	867 (20.5)	497 (12.0)
	Middle	2185 (50.9)	1511 (34.9)	674 (16.0)
	Low	737 (16.6)	509 (11.1)	228 (5.5)
Paternal education	≤ Middle school	70 (1.6)	46 (1.0)	24 (0.6)
	High school	748 (17.3)	557 (12.9)	191 (4.4)
	≥ College	1456 (34.4)	1047 (24.5)	409 (9.9)
	Unknown	2012 (46.7)	1237 (28.0)	775 (18.7)
Maternal education	≤ Middle school	50 (1.1)	39 (0.9)	11 (0.2)
	High school	906 (21.3)	675 (15.8)	231 (5.5)
	≥ College	1358 (32.0)	970 (22.6)	388 (9.4)
	Unknown	1972 (45.6)	1203 (27.2)	769 (18.4)
Self-rated health	Good	2621 (61.3)	1125 (37.5)	991 (23.8)
	Fair	1196 (28.2)	901 (21.2)	295 (7.0)
	Bad	469 (10.5)	356 (7.8)	113 (2.7)
Perceived stress	High	2154 (49.6)	1615 (36.9)	539 (12.7)
	Low	2132 (50.4)	1272 (29.6)	860 (20.8)
Experience of depression	No	2831 (66.1)	1811 (41.7)	1020 (24.4)
	Yes	1455 (33.9)	1076 (24.8)	379 (9.1)

*M* Mean, *SE* Standard error; data are presented as frequency (%) for qualitative variables and M ± SE for continuous variables

Table 2 demonstrates differences in characteristics between EWCB and non-EWCB respondents, with separate lists for girls and boys. Of the 2,887 girls, 674 (23.3%) reported EWCB; of the 1,399 boys, 162 (11.5%) reported the same. Variations in the prevalence of weight control behaviors were then investigated according to each characteristic. For girls, statistically significant effects were found for school sex-composition (*p* = 0.041), school type (*p* < 0.001), perceived stress (*p* < 0.001), and experience of depression (*p* < 0.001). For boys, statistically significant effects were observed for school type (*p* = 0.002), geographic area (*p* = 0.009), and experience of depression (*p* < 0.001).

**Table 2:** Differences in characteristics between EWCB and Non-EWCB by sex (*N* = 4286)

Variables	Categories	Girls			Boys		
		EWCB (*n* = 674) M ± SE or n (%)	Non-EWCB (*n* = 2213) M ± SE or n (%)	t or χ2 (p)	EWCB (*n* = 162) M ± SE or n (%)	Non-EWCB (*n* = 1237) M ± SE or n (%)	t or χ2 (p)
Age (years)		16.45 ± 0.04	16.51 ± 0.02	-1.38 (.168)	16.57 ± 0.08	16.57 ± 0.03	-0.06 (.955)
Sex- composition	Single-sex	277 (9.8)	998 (35.9)	4.17 (.041)	53 (3.9)	493 (35.6)	2.40 (.121)
	Co-ed	397 (13.7)	1215 (40.6)		109 (7.6)	744 (52.8)	
School type	General high	525 (18.8)	1911 (67.2)	41.07(< .001)	120 (8.7)	1024 (73.9)	9.28 (.002)
	Vocational high	149 (4.7)	302 (9.3)		42 (2.8)	213 (14.6)	
Geographic area	Big city	333 (11.6)	1157 (39.2)	0.82 (.663)	98 (6.8)	593 (40.9)	9.40 (.009)
	Mid-size/Small city	293 (10.7)	927 (34.2)		58 (4.3)	566 (43.1)	
	Rural area	48 (1.1)	129 (3.1)		6 (0.4)	78 (4.4)	
Economic status	High	201 (7.2)	666 (23.6)	0.94 (.626)	64 (4.7)	433 (31.1)	1.74 (.418)
	Middle	343 (12.0)	1168 (40.5)		71 (5.0)	603 (42.8)	
	Low	130 (4.2)	379 (12.5)		27 (1.9)	201 (14.5)	
Paternal education	≤ Middle school	11 (0.4)	35 (1.1)	4.49 (.213)	4 (0.3)	20 (1.4)	0.63 (.890)
	High school	149 (5.2)	408 (14.3)		23 (1.6)	168 (11.6)	
	≥ College	223 (8.0)	824 (28.8)		45 (3.3)	364 (26.2)	
	Unknown	291 (9.9)	946 (32.3)		90 (6.4)	685 (49.2)	
Maternal education	≤ Middle school	15 (0.5)	24 (0.7)	5.94 (.115)	2 (0.2)	9 (0.6)	2.39 (.495)
	High school	159 (5.7)	516 (18.0)		30 (2.0)	201 (14.3)	
	≥ College	219 (7.8)	751 (26.3)		44 (3.3)	344 (24.6)	
	Unknown	281 (9.5)	922 (31.5)		86 (6.1)	683 (48.9)	
Self-rated health	Good	371 (13.1)	1259 (43.2)	0.35 (.840)	108 (7.8)	883 (63.4)	1.42 (.490)
	Fair	212 (7.5)	689 (24.5)		37 (2.6)	258 (18.2)	
	Bad	91 (2.9)	265 (8.8)		17 (1.1)	96 (6.8)	
Perceived stress	High	434 (14.9)	1181 (40.5)	23.3 (< .001)	65 (4.6)	474 (33.4)	0.35 (.554)
	Low	240 (8.6)	1032 (36.0)		97 (6.9)	763 (55.1)	
Experience of depression	No	334 (11.8)	1477 (50.9)	73.2 (< .001)	94 (6.8)	926 (66.1)	16.0 (< .001)
	Yes	340 (11.7)	736 (25.6)		68 (4.8)	311 (22.3)	

*EWCB* Extreme weight control behavior, *M* Mean, *SE* Standard error; *p*-value refers to independent t-test or chi-square test

According to the sex-stratified analyses (Table 3), strong risk factors for EWCB in girls included school type, perceived stress, and experience of depression. Vocational high school attendance (vs. general high school attendance) was positively associated with EWCB (OR = 1.860, 95% CI = 1.531–2.259). The odds of EWCB were greater in cases of high perceived stress than those of low perceived stress (OR = 1.287, 95% CI = 1.057–1.566) as well as in cases of experience of depression than in cases without experience of depression (OR = 1.827, 95% CI = 1.536–2.173).

**Table 3:** Logistic regression analysis on the factors associated with EWCB by sex (*N* = 4286)

Variables	Categories	Girls (*n* = 2887)	Boys (*n* = 1399)
		OR (95% CI)	OR (95% CI)
Age (years)		0.925 (0.836–1.023)	1.004 (0.834–1.209)
Sex- composition	Single-sex	1	1
	Co-ed	1.208 (0.996–1.465)	1.355 (0.969–1.895)
School type	General high	1	1
	Vocational high	1.860 (1.531–2.259)	1.651 (1.172–2.327)
Geographic area	Big city	0.851 (0.507–1.426)	2.529 (1.053–6.075)
	Mid-size/Small city	0.879 (0.524–1.474)	1.404 (0.574–3.431)
	Rural area	1	1
Economic status	High	1.052 (0.798–1.387)	1.378 (0.837–2.267)
	Middle	0.991 (0.790–1.244)	1.077 (0.661–1.755)
	Low	1	1
Paternal education	≤ Middle school	1.005 (0.509–1.983)	1.509 (0.493–4.619)
	High school	1.242 (0.946–1.632)	1.103 (0.562–2.164)
	≥ College	1	1
	Unknown	1.150 (0.805–1.642)	1.764 (0.864–3.600)
Maternal education	≤ Middle school	1.899 (0.866–4.165)	2.327 (0.645–8.390)
	High school	0.883 (0.672–1.160)	1.030 (0.562–1.891)
	≥ College	1	1
	Unknown	0.904 (0.644–1.268)	0.586 (0.301–1.141)
Self-rated health	Good	1	1
	Fair	0.909 (0.740–1.118)	1.143 (0.743–1.759)
	Bad	0.869 (0.672–1.124)	1.317 (0.717–2.418)
Perceived stress	High	1.287 (1.057–1.566)	0.828 (0.558–1.228)
	Low	1	1
Experience of depression	Yes	1.827 (1.536–2.173)	2.232 (1.532–3.253)
	No	1	1

*EWCB* Extreme weight control behavior, *OR* Odds ratio, *CI* Confidence interval, using multiple logistic regression analysis

For males, EWCB was significantly influenced by school type, geographic area, and experience of depression. Specifically, both vocational high school attendance (vs. general high school attendance) and residence in a big city (vs. rural area) were positively and significantly associated with EWCB (OR = 1.651, 95% CI = 1.172–2.327 and OR = 2.529, 95% CI = 1.053–6.075, respectively). Similar to girls, boys who experienced depression were at higher risk for EWCB than those who had not experienced depression (OR = 2.232, 95% CI = 1.532–3.253).

## Discussion

This study investigated EWCB in a sample of high school students between 15 and 18 years of age, all of whom perceived themselves as overweight or obese despite being underweight or of normal weight. Specifically, this study focused on the clarification of which of these individuals were most prone to EWCB; in other words, the study analyzed the sample to determine the specific factors that influenced or were related to EWCB, with a further breakdown based on sex. Of the 27,919 high school students who responded to the KYRBS, nearly double the proportion of girls (vs. boys) were found to have overestimated their weight despite being underweight or of normal weight. A substantially higher proportion of girls also reported extreme diet behaviors compared to boys, at 23.3% and 11.5%, respectively. Thus, our findings support previous research that suggests that girls are more likely to overestimate their weight and engage in EWCB when compared to boys [10, 22, 23]. Non-obese adolescents who are preoccupied with a slender body prefer extreme weight-loss methods with immediate effects rather than the improvement of their eating and exercise habits [24]. There is a considerable need to provide interventions to help adolescents develop accurate perceptions of their body weight or correct their already distorted weight perceptions.

In terms of the associated factors, regardless of EWCB engagement, vocational high school attendance imposed a significant influence across the analyzed subpopulation. In a previous study, Park, Woo, and Her [25] found that female students who attended vocational high schools reported higher levels of concern over their appearance and demonstrated a higher prevalence of eating disorders than those at academically oriented general high schools. In a similar study, vocational high school students reported more body image distortion than academically oriented general high school students [26].

In South Korea, several companies continue to engage in appearance-discriminatory hiring practices, which thus induces high and continually increasing anxiety among job seekers [27]. According to a 2021 report issued by the National Youth Policy Institute, vocational high school students (vs. general high school students) are significantly more concerned about experiencing discrimination based on their appearance or physical condition, including their height and weight [4]. While research on appearance discrimination in the job market has traditionally focused on girls, new scholarly evidence [28, 29] and media reports [30, 31] suggest that boys also face this problem.

The Ministry of Education recommends that obese adolescents screened through health checkups in a school environment be educated on how to improve their eating habits and physical activities. However, this is not adequately enforced due to the low priority of health-related education in the university entrance exam-oriented educational environment of Korea [32, 33]. So far, the Korean government has not been particularly interested in policy that regards gendered appearance stereotypes and appearance obsession prevalent in Korean society. The key to changing this phenomenon (culture) is to change societal perceptions [33]. Adolescents spend most of their time in school. Schools are optimized environments that can motivate adolescents' health promotion behavior and, can serve as intervention points that can aid the changing of their perceptions. A school's obesity intervention approach that targets obese adolescents only based on the BMI alone cannot correct the subjective body type perception of non-obese adolescents, and there is a risk of overlooking the opportunity to improve health promotion behaviors [2, 33].

The School Health Act, one of the main areas of youth life, specifies that health education should be conducted. This provision can be the basis for the use of health education as a means of improving adolescent health [33]. Respect for the diversity of adolescents' appearance and body is very important in daily life, and related education is very essential [34]. For example, the National Eating Disorders Association (NEDA) in the U.S. recommends that children and adolescents eat a balanced diet and exercise rather than diet to lose weight, and above all, emphasizes that they not define or discriminate against each other's appearance and body, and respect various appearances and bodies [35].

Additionally, the school site should be thoroughly evaluated and monitored for changes, such as whether students’ appearance distortion or EWCB is being improved through related education. Vocational high schools, especially, provide field practice-oriented education for the purpose of training human resources to engage in specific jobs [4]. Compared to general high school students, these students are more likely to experience difficulties in the early transition to the labor market where appearance premiums exist [36]. Education should be actively conducted so that vocational high school students can fully learn about body and appearance-related discourse. A differentiated health education program should be provided so that sufficient discussion among students can be conducted and implemented through relevant activities.

After overcoming various obstacles, the Law of the Fair Hiring Procedure Act was passed in 2019. However, this did not eliminate the practice of the requirement of headshot photographs of job applicants. In this context, photographs are considered key visual elements for the identification of the gender and appearance of applicants and may thus be used in discriminatory hiring practices [27]. Despite the expansion of blind recruitment, a recent survey found that eight out of 10 companies required applicants to attach photographs to their resumes, with 62.6% reporting the elimination of applicants based on photographs while screening documents [37]. In this regard, Lim argues that blind recruitment should be legislatively mandated, as applicants are unfairly expected to seek employment from private companies that uphold cultures of appearance discrimination at the moment [28].

Adolescence is the second growth period; the pace of growth and development is fast, physical and sexual maturity related to secondary sexual characteristics progresses rapidly, and the individual experiences changes in appearance. The body is self-representation for adolescents [38]. Teenagers uncritically internalize their socially and culturally idealized bodies [2] and compare their appearance with their peer group's physical development or ideal form of thinking for themselves, due to which they sometimes experience embarrassment or anxiety [38]. Unlike general high school students, vocational high school students are required by their curriculum to conduct industrial field practices to improve their field-based skills as professional workers [39]. Therefore, they are more likely to be exposed to appearance discrimination that was prevalent in the job market and form a tendency to control their weight in order to have an appearance that meets social standards [40].

We also found that experiences of depression were associated with increased odds of EWCB for all analyzed respondents, which is consistent with existing evidence [10, 14, 21, 22]. For example, studies have demonstrated that depression during adolescence predicts EWCB and is associated with binge eating/purging in young adulthood [41, 42]. Meanwhile, mental health is more strongly related to subjective weight perceptions than objective weight [43]. Feeling too fat, and especially obese was associated with worse mental health outcomes [43]. Body image distortion or body image dissatisfaction was a predictor of depression not only for female adolescents but also for male adolescents [44, 45]. In this context, depression is deeply linked to EWCB and/or dieting in non-overweight adolescents [10, 46]. In sum, these results underline the importance of screening all adolescents for depression, regardless of their weight [22, 47].

We found that perceived stress was a significant EWCB risk factor only for girls. This result reinforces a 2020 social survey conducted by Statistics Korea, which found that the second-most prevalent worry for Korean adolescents was appearance (studying was first), wherein girls reported more appearance-related stress than boys [48]. Among older adolescents (particularly girls), those with normal weight became increasingly associated with greater weight and shape concern [49]. Additionally, female adolescents had higher body dissatisfaction and were more vulnerable to sociocultural pressures than male adolescents [50]. These previous studies [49, 50] supported the notion that girls tend to endure more social pressure to maintain skinny physiques that reflect the standard ideal body type. Consequently, girls are at higher risk of EWCB, as such behaviors are often induced by stress over one’s appearance, concerns about obesity, and/or weight loss aims [11, 47, 51].

For boys, living in a big city was a significant EWCB risk factor. Story et al. [52] found few appreciable location (urban, sub-urban, or rural) differences in the prevalence of chronic dieting for either boys or girls, which suggested that these behaviors have permeated all levels of locations of residence. This inconsistent result may be due to differences in research participants or sociocultural backgrounds, so further consideration through repeated research is needed. According to a study by Gonzaga et al. [53], boys’ dissatisfaction with being overweight increased in 2017/2018 compared to 2007, which demonstrated a higher pattern than dissatisfaction with being thin. There was no gender difference in weight bias internalization, which indicated the degree of negative, prejudicial attitudes toward persons with obesity [54]. Compared to rural areas, it was found that boys who lived in big cities may adopt EWCBs to attain male beauty/attractiveness ideals (e.g., lean, and muscular body) due to greater social pressure from peers and mass media [55, 56].

## Limitations

This study had some limitations. First, self-reported data such as height and weight were used, which may entail respondent bias. Second, the cross-sectional characteristics of the data precluded the establishment of causal relationships. Finally, the survey measured multiple constructs based on single questions to assess levels of stress or depression. Despite these limitations, this study had several strengths, including the selection of study participants from a national sample representing Korean high school students. In addition, the findings of this study can provide better insight and aid in the development of school obesity prevention programs by explaining the risk factors for EWCB by sex among non-obese high school students.

## Conclusions

It is not uncommon for adolescents to overestimate their weight. Among those who do, some will ultimately choose to engage in EWCBs, which are deleterious to health and development. To clarify this problem, this study aimed to reveal the factors and characteristics that were most strongly associated with such behaviors by using data that represented Korean high school students. Results revealed that attending vocational high schools and the experience of depression were significantly associated with extreme diet behaviors, regardless of gender.

EWCB was associated with perceived stress in girls, and living in a big city in boys.

Our study’s findings suggest the importance of the provision of quality health education, including that for non-obese adolescents, in school obesity prevention programs along with the expansion of tailored intervention programs based on sex, following the consideration of the characteristics of high schools as well as individuals.

## Data Availability

The datasets generated and/or analyzed during the current study are available in the KCDC repository, https://www.kdca.go.kr/yhs/.

## Abbreviations

EWCB: Extreme weight control behavior
KYRBS: Korea Youth Risk Behavior Survey
KCDC: Korea Centers for Disease Control and Prevention
OR: Odds ratio
CI: Confidence interval
M: Mean
SE: Standard error

## References

[1] Patte KA, Livermore M, Qian W, Leatherdale ST (2021). Do weight perception and bullying victimization account for links between weight status and mental health among adolescents?. BMC Public Health.

[2] Joeng EH, Lee IS (2018). Multilevel analysis of factors associated with subjective weight perception among normal body weight adolescents based on the 2017 Korean Youth’s Risk Behavior Survey (KYRBS). J Korean Acad Comm Health Nurs.

[3] Talamayan KS, Springer AE, Kelder SH, Gorospe EC, Joye KA (2006). Prevalence of overweight misperception and weight control behaviors among normal weight adolescents in the United States. Sci World J.

[4] Kim Y, Austin SB, Subramanian SV, Thomas JJ, Eddy KT, Franko DL, Rodgers RF, Kawachi I (2018). Risk factors for disordered weight control behaviors among Korean adolescents: multilevel analysis of the Korea Youth Risk Behavior Survey. Int J Eat Disord.

[5] Tuffa TA, Gebreyesus SH, Endris BS, Getnet Y, Abebe DS (2020). Unhealthy weight control behaviors among Ethiopian female adolescents. Int J Eat Disord.

[6] Johnson ER, Weiler RM, Barnett TE, Pealer LN (2016). Extreme weight-control behaviors and suicide risk among high school students. J Sch Health.

[7] Lampard AM, Maclehose RF, Eisenberg ME, Larson NI, Davison KK, Neumark-Sztainer D (2016). Adolescents who engage exclusively in healthy weight control behaviors: who are they?. Int J Behav Nutr Phys Act.

[8] Levinson JA, Sarda V, Sonneville K, Calzo JP, Ambwani S, Austin SB (2020). Diet pill and laxative use for weight control and subsequent incident eating disorder in US young women: 2001–2016. Am J Public Health.

[9] Neumark-Sztainer D, Wall M, Larson NI, Eisenberg ME, Loth K (2011). Dieting and disordered eating behaviors from adolescence to young adulthood: findings from a 10-year longitudinal study. J American Dietetic Assoc.

[10] Nagata JM, Garber AK, Tabler JL, Murray SB, Bibbins-Domingo K (2018). Differential risk factors for unhealthy weight control behaviors by sex and weight status among U.S. adolescents. J Adolesc Health.

[11] Kim M, Lee H (2010). Overestimation of own body weights in female university students: associations with lifestyles, weight control behaviors and depression. Nutri Resd Pract.

[12] Das JK, Salam RA, Thornburg KL, Prentice AM, Campisi S, Lassi ZS, Koletzko B, Bhutta ZA (2017). Nutrition in adolescents: physiology, metabolism, and nutritional needs. Ann N Y Acad Sci.

[13] Kennedy AK, Schneiderman JU, Winter VR (2019). Association of body weight perception and unhealthy weight control behaviors in adolescence. Child Youth Serv Rev.

[14] Gonsalves D, Hawk H, Goodenow C (2014). Unhealthy weight control behaviors and related risk factors in Massachusetts middle and high school students. Matern Child Health J.

[15] Gu YJ, Jeong JY, Jeong JY, Lee HJ (2019). Comparison of factors affecting weight control experiences by perception types of body shape. Korean J Health Educ Promot.

[16] Oh DN, Kim EM, Kim S (2013). Weight control behaviors and correlates in Korean adolescents. J Korea Contents Assoc.

[17] Park S, Kim JI (2016). Latent class analysis of the weight-control behaviors in adolescents. Youth Facil Environ.

[18] Kim MO, Chang UJ (2009). A study on the perception of obesity by age and the attitude toward weight control. Korean J Food Nutr.

[19] Ministry of Education, Ministry of Health and Welfare, Korea Centers for Disease Control & Prevention (2019). The 15th Korea Youth Risk Behavior Survey Statistics.

[20] Kim Y, Choi S, Chun C, Park S, Khang YH, Oh K (2016). Data resource profile: the Korea Youth Risk Behavior Web-based Survey (KYRBS). Int J Epidemiol.

[21] Weng CB, Sheu JJ, Chen HS. Factors associated with unhealthy weight control behaviors among a representative sample of U.S. high school students. J Sch Nurs. 2020;1059840520965497. 10.1177/1059840520965497. 10.1177/1059840520965497 33073668

[22] Han J, Kim S, Park CG (2020). Gender differences in risk factors influencing unhealthy weight control behaviors among adolescents. West J Nurs Rese.

[23] Kim JS, Seo Y (2021). Associations between weight perception, unhealthy weight control behavior, and suicidal ideation and planning among Korean adolescents: a national cross-sectional secondary analysis. J Pediatr Nurs.

[24] Fan M, Jin Y (2015). The effects of weight perception on adolescents’ weight-loss intentions and behaviors: evidence from the Youth Risk Behavior Surveillance Survey. Int J Environ Res Public Health.

[25] Park HJ, Woo MO, Her ES (2015). Influence of eating disorder, anthropometric characteristics and concern in appearance-on-appearance management behavior in high school female students: focusing on the difference between general and beauty specialized high school. J Korean Soc Cosmetol.

[26] Park EJ, Kim NS (2014). Obesity and underweight among Korean women. Health Welfare Forum.

[27] Lim IS (2019). Discussion on legalization of photo-information abolition for preventing discrimination against women – appearance in the recruitment process. Korean Assoc Women’s Stud.

[28] Park G, Kim S, Son H, Lee Y, Lee H, Jung R (2011). Survey on discriminatory practices in corporate recruitment processes.

[29] Yang HK, Kim JY (2015). The effect of obesity in the recruiting market: the case of young Korean college graduates. Kor Health Econ Rev.

[30] Lee S. Ability has nothing to do with appearance. Yonhap News Agency. 2017. https://www.yna.co.kr/view/AKR20170516158500797?section=search. Accessed 6 Apr 2022.

[31] Lee Y. More than half of the companies, the appearance of applicants affects the employment evaluation! Financial Today. 2020. http://www.ftoday.co.kr/news/articleView.html?idxno=208289. Accessed 6 Apr 2022.

[32] Kim H, Cho J, Kim S, Kang Y (2014). A study on the preparation of obesity prevention measures for children and youth.

[33] Im H, Baek H, Kim D (2019). A study on policy plans for youth health right.

[34] Kim D, Kim Y, Dong J, Jeong D, Kim S (2019). Gender and health inequality in Korea: focusing on body obsession and aesthetic plastic surgery.

[35] NEDA (2022). Developing modeling positive body image.

[36] Sierminska E, Singhal K. Does it pay to be beautiful? IZA World Labor. 2023;161v2. 10.15185/izawol.161.v2.

[37] Lee J. Despite the expansion of “blind recruitment” . . . 8 out of 10 businesses require resume photos. Edaily. 2019. https://www.edaily.co.kr/news/read?newsId=02007366622658496&mediaCodeNo=257&OutLnkChk=Y. Accessed 6 Apr 2022.

[38] Korean Academy of Child and Adolescent Psychiatry (2012). Adolescent psychiatry.

[39] Ministry of Education. The Ministry of Education supports safe field training and employment of vocational high school graduates. 2021. https://www.moe.go.kr/boardCnts/viewRenew.do?boardID=295&lev=0&statusYN=W&s=moe&m=020401&opType=N&boardSeq=84175.

[40] Kim Y (2013). Sociocultural influence on appearance as appearance management attitude among adolescent girls and boys. J Kor Soc B & A.

[41] Liechty JM, Lee MJ (2013). Longitudinal predictors of dieting and disordered eating among young adults in the U.S. Int J Eat Disord.

[42] Stephen EM, Rose JS, Kenney L, Rosselli-Navarra F, Weissman RS (2014). Prevalence and correlates of unhealthy weight control behaviors: findings from the national longitudinal study of adolescent health. J Eat Disord.

[43] Jansen WM, van de Looij-Jansen PMM, de Wilde EJ, Brug J (2008). Feeling fat rather than being fat may be associated with psychological well-being in young Dutch adolescents. J Adolesc Health.

[44] Stice E, Bearman SK (2001). Body-image and eating disturbances prospectively predict increases in depressive symptoms in adolescent girls: a growth curve analysis. Dev Psychol.

[45] Blashill AJ, Wilhelm S (2014). Body image distortions, weight, and depression in adolescent boys: longitudinal trajectories into adulthood. Psychol Men Masc.

[46] Crow S, Eisenberg ME, Story M, Neumark-Sztainer D (2006). Psychosocial and behavioral correlates of dieting among overweight and non-overweight adolescents. J Adolesc Health.

[47] Lee KM, Seo MS, Shim JY, Lee YJ (2015). Body weight status misperception and its association with weight control behaviours, depressive mood and psychological distress in nulliparous normal-weight young women. Ann Hum Biol.

[48] Statistics Korea (2020). Social survey: worries of the youths (main response, 13–18 years old).

[49] Calzo JP, Sonneville KR, Haines J, Blood EA, Field AE, Austin SB (2012). The development of associations among body mass index, body dissatisfaction, and weight and shape concern in adolescent boys and girls. J Adolesc Health.

[50] Esnaola I, Rodríguez A, Goñi A (2010). Body dissatisfaction and perceived sociocultural pressures: gender and age differences. Salud Ment.

[51] Bašková M, Holubčíková J, Baška T (2017). Body-image dissatisfaction and weight-control behaviour in Slovak adolescents. Central European J Pub Health.

[52] Story M, Rosenwinkel K, Himes JH, Resnick M, Harris LJ, Blum RW (1991). Demographic and risk factors associated with chronic dieting in adolescents. Am J Dis Child.

[53] Gonzaga I, Ribovski M, Claumann GS, Folle A, Beltrame TS, Laus MF, Pelegrini A (2023). Secular trends in body image dissatisfaction and associated factors among adolescents (2007–2017/2018). PLOS ONE.

[54] Puhl RM, Himmelstein MS (2018). Weight bias internalization among adolescents seeking weight loss: implications for eating behaviors and parental communication. Front Psychol.

[55] McCabe MP, Ricciardelli LA, Sitaram G, Mikhail K (2006). Accuracy of body size estimation: role of biopsychosocial variables. Body Image.

[56] Xie B, Chou CP, Spruijt-Metz D, Reynolds K, Clark F, Palmer PH, Gallaher P, Sun P, Guo Q, Johnson CA (2006). Weight perception and weight-related sociocultural and behavioral factors in Chinese adolescents. Prev Med.

